# Causal relationship between gut microbes and cardiovascular protein expression

**DOI:** 10.3389/fcimb.2022.1048519

**Published:** 2022-12-05

**Authors:** Wenchuan Zhang, Shuwan Zhang, Feng Zhao, Jinda Du, Zhe Wang

**Affiliations:** ^1^ Department of Pathology, Shengjing Hospital of China Medical University, Shenyang, Liaoning, China; ^2^ Department of Stem Cells and Regenerative Medicine, Shenyang Key Laboratory of Stem Cell and Regenerative Medicine, China Medical University, Shenyang, Liaoning, China; ^3^ Department of Gastroenterology, General Hospital of Northern Theatre Command, Shenyang, Liaoning, China

**Keywords:** gut microbes, cardiovascular proteins, Mendelian randomization, causal effect, genome-wide association study

## Abstract

Evidence supports associations between gut microbiota and cardiovascular protein levels in plasma. However, it is unclear whether these associations reflect a causal relationship. To reveal the causal relationship between gut microbiota and cardiovascular protein levels in plasma, we estimated their causal effects using two-sample Mendelian randomization (MR) analysis. Sensitivity analysis was also performed to assess the robustness of our results. Genome-wide association study (GWAS) of microbiomes in the MiBioGen study included 211 bacterial taxa (18,473 individuals), and GWAS of 90 cardiovascular proteins included 30,931 individuals. There were 196 bacterial taxa from five levels available for analysis. The following 14 causal relationships were identified: phylum *Euryarchaeota* and carbohydrate antigen 125 (β = 0.289), order *Bacillales* and CSF-1 (β = -0.211), genus *Dorea* and HSP-27 (β = 0.465), phylum *Actinobacteria* and IL-8 (β = 0.274), order *Enterobacteriales* and KIM-1 (β = -0.499), class *Actinobacteria*, genus *Bifidobacterium*, phylum *Actinobacteria* and LEP (β = -0.219, β = -0.201, and β = -0.221), genus *Methanobrevibacter* and NT-proBNP (β = 0.371), family *Peptostreptococcaceae* and SRC (β = 0.191), order *Verrucomicrobiales*, phylum *Verrucomicrobia* and TNF-R2 (β = 0.251 and β = 0.233), family *Veillonellaceae* and t-PA (β = 0.271), and class *Erysipelotrichia* and VEGF-D (β = 0.390). Sensitivity analysis showed no evidence of pleiotropy or heterogeneity. The results of the reverse MR analysis showed no reverse causality for any of the 13 gut microbes and 11 cardiovascular proteins. Mendelian randomization estimates provide strong evidence for a causal effect of gut microbiota-mediated alterations on cardiovascular protein expression.

## Introduction

Gut microbiota is a dynamic and complex ecological community of microorganisms that inhabit the human gut (O’Hara and Shanahan, 2006). Twin-based heritability estimates and macrogenome-wide association studies have challenged the conventional view of the gut microbiota as a purely environmental factor (Blekhman et al., 2015; Turpin et al., 2016; Liu et al., 2021). Gut microbiota affects immunity, neurology, psychiatric traits, metabolism, and even circulation of the body (Gilbert et al., 2018). Many disease states have been associated with changes in faecal microbiota, including cardiovascular disease (Brown and Hazen, 2015). Indeed, specific gut microbiota-dependent metabolites affect host metabolism and cardiovascular disease. For example, phenylacetyl glutamine promotes adverse cardiovascular phenotypes in the host by interacting with multiple adrenergic receptors (Nemet et al., 2020). Additionally, it has been suggested that gut microbiota directly influence plasma levels of proinflammatory factors including some cardiovascular proteins. For example, Lee et al. found that enterotoxigenic *Bacteroides* fragilis-secreted toxin induces IL-8 secretion from intestinal epithelial cells *via* the E-Cadherin/β-Catenin/NF-κB pathway (Lee et al., 2022). Engevik et al. found that *F. nucleatum* produces outer membrane vesicles that activate Toll-like receptor 4 to drive extracellular signal-regulated kinase, NF-κB, and proinflammatory cytokine expression (Engevik et al., 2021). Pallikkuth et al. studied rhesus monkeys as non-human primates, and found that highly abundant archaea and amoebae in aged animals had a direct correlation with plasma biomarkers of inflammation and immune activation, such as interleukin (IL)-6, IL-8, and TNF (Pallikkuth et al., 2021). However, most studies have examined only a few gut microbes and cardiovascular proteins. No comprehensive or systematic randomized controlled trials have been conducted on the causal relationship between gut microbes and cardiovascular protein expression in humans.

Typically, the gold standard to infer causality is a randomized controlled trial (RCT). Considering the difficulty in implementing RCTs, Mendelian randomization (MR) takes full advantage of this method. It is an epidemiological method that strengthens chance inferences by genetic variation as an instrumental variable for exposure (Burgess and Thompson, 2015). Because parental alleles are randomly assigned to offspring during meiosis in accordance with Mendel’s second law, the method minimizes the interference of important confounding factors such as the natural environment and socioeconomic level. The use of genetic variation as a valid instrumental variable in MR analysis relies on three core assumptions. First, genetic variation must be strongly correlated to exposure factors (association assumption). Second, genetic variation cannot be directly correlated to outcomes (exclusion restriction assumption). Third, genetic variation cannot be correlated to any possible confounding factors (independence assumption) (Emdin et al., 2017). To date, genetic studies have shown that host genetic variation affects the composition of the gut microbiota (Sanna et al., 2019). Therefore, in the present study, we performed two-sample MR analysis to fully explore whether gut microbiota abundance has a causal effect on cardiovascular protein levels and to identify specific pathogenic bacterial taxa.

## Material and methods

### Gut microbial samples

Sample summary statistics employed GWAS meta-analysis of the gut microbiome in the MiBioGen study, the largest cohort, multi-ethnic, genome-wide gut microbiome meta-analysis to date. The meta-analysis included 18,340 individuals from 22 cohorts consisting of adults or adolescents and two cohorts consisting of children. There were four multiple ancestry samples. Single ancestry samples included 16 European cohorts, one Middle Eastern cohort, one East Asian cohort, one US Hispanic/Latin cohort, and one African American cohort. The microbial composition was analysed by targeting three different variable regions of the 16S rRNA gene: V1–V2 (n=3,716), V3–V4 (n=4,211), and V4 (n=10,413). A total of 211 taxa were included in this study (131 genuses, 35 families, 20 orders, 16 phyla, and nine classes) (Kurilshikov et al., 2021).

### Cardiovascular protein samples

Cardiovascular protein phenotypes were obtained from a recent genomic analysis of 90 cardiovascular proteins in 30,931 individuals. The study included 15 cohorts (13 discovery datasets and two replication datasets). The analysis identified 451 protein quantitative trait loci associated with the plasma levels of 85 proteins (*P<* 5×10^-8^) (Folkersen et al., 2020).

### Genetic instrument selection

Bacterial taxa were analysed at five levels (phylum, class, order, family, and genus). A unique taxonomic unit was defined as a feature. Considering that a gene cis region only constitutes a small proportion of the genome, we relaxed the conventional genome-wide significance P-value threshold for instrument selection to P<1×10^-5^ (Sanna et al., 2019). We implemented a series of additional quality control steps to select eligible instrumental variables (IVs). First, SNPs with allelic inconsistencies between the exposure and result samples (i.e., A/G vs. A/C) were excluded. Second, palindromic A/T or G/C alleles were excluded to avoid distortions in strand orientation or allelic coding. Third, SNPs within each bacterial taxonomic unit were clustered together and only independent SNPs were retained. The linkage disequilibrium (LD) threshold for clustering was set to r2< 0.01 and the size of the clustering window was set to 500 kb. A total of 1,000 Genome Project sequencing data (phase 3) were used to estimate LD. Fourth, horizontal pleiotropy effects, i.e., confounding effects caused by other diseases, may violate the second hypothesis in the MR analysis (SNPs are not associated with outcomes). Once horizontal pleiotropic effects were found, this instrumental variable was deleted. The MR-Egger regression test and MR-PRESSO test were applied to detect potential horizontal pleiotropy and to eliminate the effect of pleiotropy by removing outliers (Verbanck et al., 2018). Additionally, genomic coordinates for all correlation analyses were based on Ensembl GRCh37 reference during the coordination process (Yates et al., 2020), which removed ambiguous and duplicate SNPs. For markers without SNP information, SNP conversion was performed using the ieugwasr package for chr:pos (radius set to 0).

### Mendelian randomization analysis

We performed MR analyses to investigate the causal relationships between gut microbiota and 90 cardiovascular proteins. The causal relationship between each pair of bacterial taxa and plasma metrics was examined using four MR methods: the inverse variance weighted (IVW) test (Burgess et al., 2013), weighted median estimator (WME) (Bowden et al., 2016), MR-Egger regression (Bowden et al., 2015), and MR-PRESSO (Verbanck et al., 2018). Each of the four statistical methods has its own model assumptions that are explained in detail in each study (Burgess et al., 2013; Bowden et al., 2015; Bowden et al., 2016; Verbanck et al., 2018). The IVW method is slightly stronger than the other methods under certain conditions (Bowden et al., 2016). Therefore, we performed MR estimation using IVW as the main method. To account for multiple testing, we implemented a Bonferroni-corrected allowed type I error rate (α) of 0.05/n (the effective number of independent bacterial taxa at the taxonomic level): phylum P = 5.56 × 10^-3^ (0.05/9), class P = 3.33 × 10^-3^ (0.05/15), order P=2.50×10^-3^(0.05/20), family P=1.56×10^-3^ (0.0 5/32), and genus P=4.20×10^-4^ (0.05/119). To assess the robustness of significant results, we performed several sensitivity analyses. The Cochrane Q test for IVW and MR-Egger regression was used to test for potential heterogeneity. Moreover, leave-one-out analyses were performed to determine whether the causal signal was driven by a single SNP. Additionally, to assess the strength of the selected SNPs, the F-statistic was calculated for each bacterial taxonomic unit using the following equation:


$$F=\frac{R^{2}(n-1-k)}{(1-R^{2})k}$$

where R^2^ is the fraction of exposed variance explained by IVs, k is the number of IVs, and n is the sample size. F-statistic ≥ 10 indicates no strong evidence of weak instrument bias. IVs with an F-statistic of less than 10 were considered weak IVs and excluded (Pierce et al., 2011). Because the minor allele frequency was not provided for SNPs in gut microbes, we estimated the R^2^ values directly using the get_r_from_pn function of the TwoSampleMR package. Additionally, we performed reverse MR analysis to explore the reverse causality of cardiovascular proteins. MR analysis was performed in R (version 4.1.3) (http://www.r-project.org ) using the TwoSampleMR package (Hemani et al., 2018).

## Results

**Figure 1:**
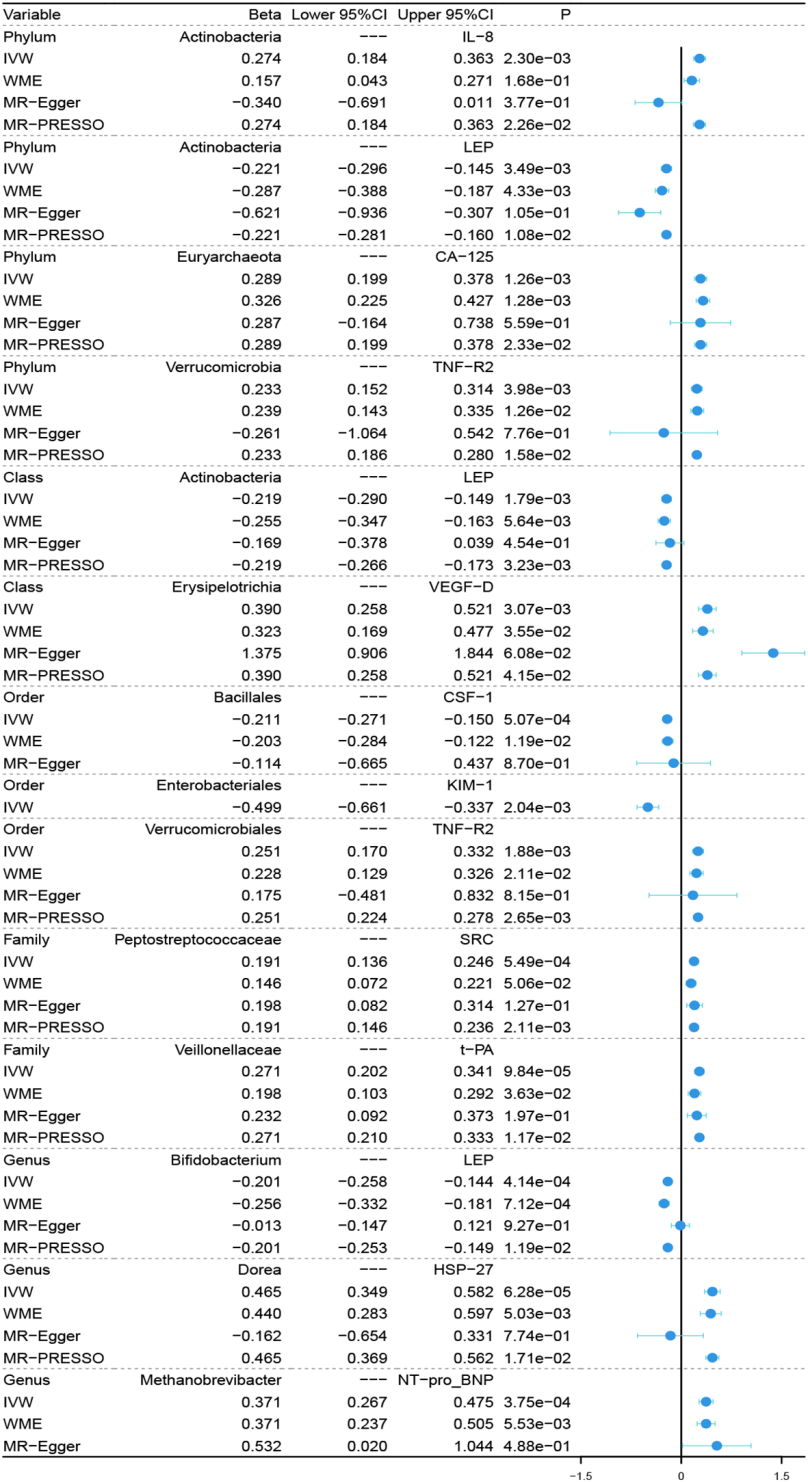
Forest plot of causal relationships estimated for 13 microbiota and 11 cardiovascular proteins using four MR methods. IVW, inverse variance weighted; WME, weighted median estimator; MR-Egger, MR-Egger regression; MR-PRESSO, MR-PRESSO test; MR, Mendelian randomization.

**Figure 2:**
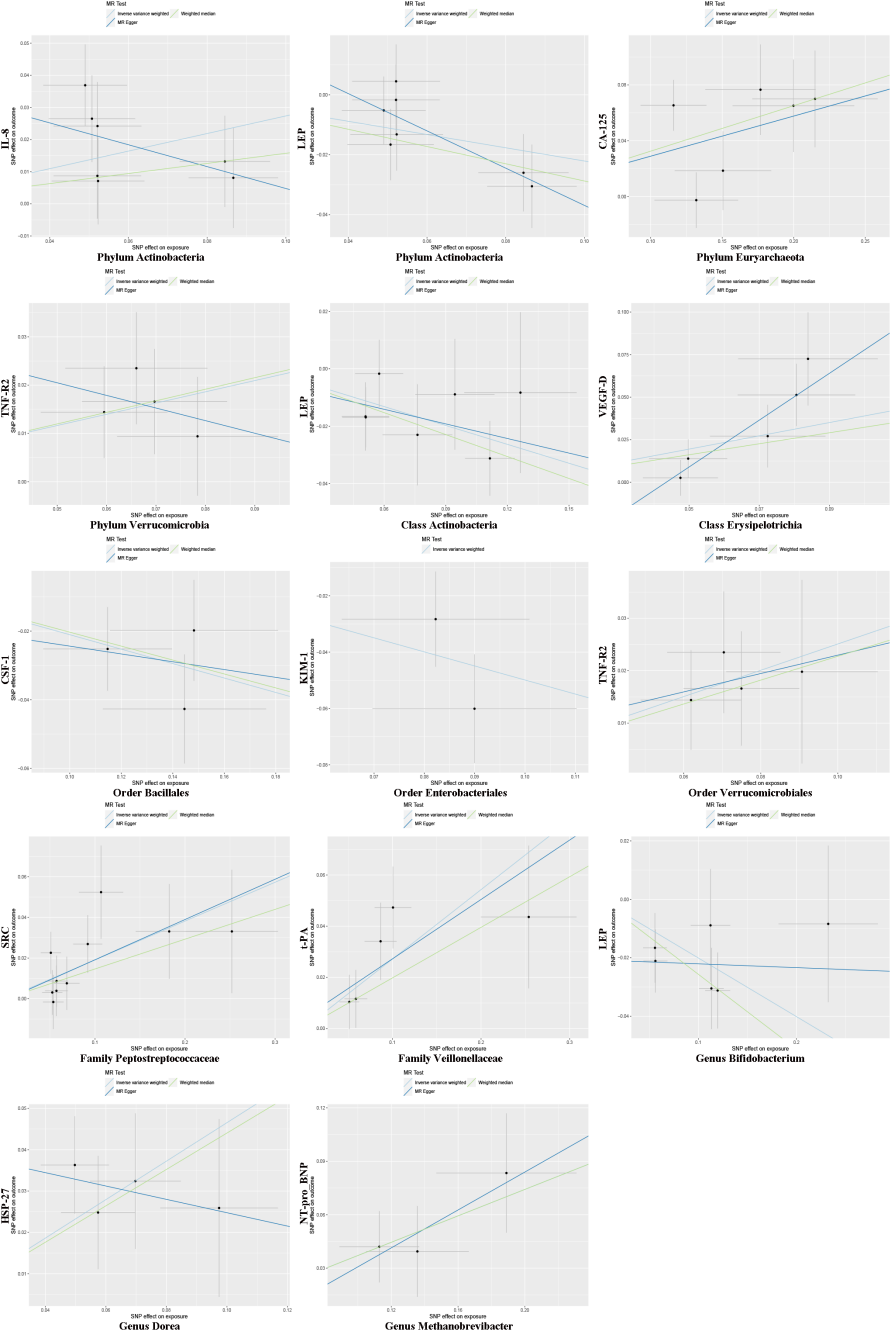
Mendelian randomization estimation using multiple SNPs. Light blue, green, and dark blue lines represent the association between microbiota (exposure) and cardiovascular proteins (outcome) estimated by the IVW method, weighted median estimator, and MR-Egger regression, respectively. Vertical and horizontal black lines around each point show the 95% confidence interval for each polymorphism exposure association and polymorphism outcome association, respectively. SNP, single nucleotide polymorphism.

In the microbiome samples, we excluded three unknown families and 12 unknown genuses. Additionally, we found duplicate data for class *Verrucomicrobiae* and order *Verrucomicrobiales*, but fortunately, this did not affect the results. We therefore chose to retain the order *Verrucomicrobiales* with lower species relationships. Thus, nine phyla, 15 classes, 20 orders, 32 families, and 131 genuses bacterial taxa were included in the subsequent MR analysis. MR analyses by the IVW test are summarized in **Supplementary Tables 1–3**. We found 14 causal relationships between 13 bacterial characteristics and 11 cardiovascular protein indicators, including phylum *Euryarchaeota* and carbohydrate antigen 125 (CA-125) (P = 1.26 × 10^-3^), order *Bacillales* and colony-stimulating factor (CSF-1) (P = 5.07 × 10^-4^), genus *Dorea* and heat shock protein 27 (HSP-27) (P = 6.28 × 10^-5^), phylum *Actinobacteria* and interleukin-8 (IL-8) (P = 2.30 × 10^-3^), order *Enterobacteriales* and kidney injury molecule-1 (KIM-1) (P = 2.04 × 10^-3^), class *Actinobacteria*, genus *Bifidobacterium*, and the phylum *Actinobacteria* and leptin (LEP) (P = 1.79 × 10^-3^, P = 4.14 × 10^-4^, and P = 3.49 × 10^-3^, respectively), genus *Methanobrevibacter* and N-terminal pro-brain natriuretic peptide (NT-proBNP) (P = 3.75 × 10-4), family *Peptostreptococcaceae* and proto-oncogene tyrosine-protein kinase Src (SRC) (P = 5.49 × 10^-4^), order *Verrucomicrobiales* and phylum *Verrucomicrobia*, and tumor necrosis factor receptor 2 (TNF-R2) (P = 1.88×10^-3^ and P = 3.98×10^-3^, respectively), family *Veillonellaceae* and tissue plasminogen activator (t-PA) (P = 9.84 × 10^-5^), and class *Erysipelotrichia* and vascular endothelial growth factor D (VEGF-D) (P = 3.07 × 10^-3^). Among them, nine bacterial traits were positively correlated to IL-8, CA-125, TNF-R2, VEGF-D, SRC, t-PA, HSP-27, and NT-proBNP with regression coefficients ranging from 0.19 to 0.47. Five bacterial traits were negatively and causally correlated to LEP, CSF-1, and KIM-1 with regression coefficients ranging from -0.50 to -0.20. Notably, the causal effects of phylum and class *Actinobacteria* on LEP, and phylum and order *Verrucomicrobia* on TNF-R2 were consistent in direction (**Figure 1**). Scatter plots of the 14 causal IVW, WME, and MR-Egger are shown in **Figure 2**. The F-statistics for all IVs were greater than 10, indicating no evidence of weak instrument bias (**Supplementary Table 4**). The MR-Presso test and MR-Egger regression test did not show a horizontal multidirectional effect (p>0.05), whereas the statistics for the IVW test and MR-Egger regression showed no evidence of heterogeneity among the identified outcomes (P>0.05) (**Table 1**). Additionally, the leave-one-out sensitivity analysis showed that no single SNP significantly affected the causal association (**Supplementary Figure 1**). The reverse MR analysis showed no common SNPs for cardiovascular proteins (P< 1 × 10^-5^) and the identified bacterial features. Therefore, we did not find the presence of reverse causality.

**Table 1:** Sensitive analysis of identified bacterial taxa with cardiovascular proteins.

Bacterial taxa(exposure)	cardiovascular proteins (outcome)	No. of SNP	Pleiotropy					Heterogeneity		
			MR-PRESSO Global P-value	Egger intercept	intercept’s se	MR-egger P-value	IVW test’s Q	IVW test P-value	MR Egger’s Q	MR-egger P-value
Phylum *Actinobacteria*	IL-8	7	0.401	0.039	0.022	0.131	6.770	0.343	3.523	0.620
Phylum *Actinobacteria*	LEP	7	0.726	0.025	0.019	0.247	3.850	0.697	2.133	0.831
Phylum *Euryarchaeota*	CA-125	6	0.191	0.000	0.068	0.997	8.735	0.120	8.735	0.068
Phylum *Verrucomicrobia*	TNF-R2	4	0.829	0.034	0.054	0.599	1.014	0.798	0.632	0.729
Class *Actinobacteria*	LEP	7	0.875	-0.004	0.016	0.810	2.619	0.855	2.554	0.768
Class *Erysipelotrichia*	VEGF-D	5	0.324	-0.060	0.028	0.119	5.831	0.212	1.157	0.763
Order *Bacillales*	CSF-1	3	NA	-0.013	0.074	0.889	1.205	0.547	1.169	0.280
Order *Enterobacteriales*	KIM-1	2	NA	NA	NA	NA	1.189	0.276	NA	NA
Order *Verrucomicrobiales*	TNF-R2	4	0.957	0.005	0.047	0.918	0.337	0.953	0.323	0.851
Family *Peptostreptococcaceae*	SRC	10	0.797	-0.001	0.009	0.947	5.922	0.748	5.917	0.656
Family *Veillonellaceae*	t-PA	5	0.579	0.004	0.012	0.768	3.131	0.536	3.026	0.388
Genus *Bifidobacterium*	LEP	6	0.546	-0.021	0.013	0.197	4.182	0.523	1.796	0.773
Genus *Dorea*	HSP-27	4	0.651	0.041	0.031	0.320	2.079	0.556	0.362	0.834
Genus *Methanobrevibacter*	NT-pro_BNP	3	NA	-0.022	0.070	0.802	0.341	0.843	0.238	0.626

IL-8, Interleukin-8. LEP, Leptin. CA-125, Carbohydrate antigen 125. TNF-R2, Tumor necrosis factor receptor 2. VEGF-D, Vascular endothelial growth factor D. CSF-1, Macrophage-colony stimulating factor. KIM-1, Kidney injury molecular1. SRC, Proto-oncogene tyrosine-protein kinase Src. t-PA, Tissue plasminogen activator. HSP-27, Heat shock protein 27. NT-pro_BNP, N-terminal pro-brain natriuretic peptide. NA, Not Applicable. No. of SNP, Number of SNPs.

## Discussion

In this study, we performed MR analyses to assess the potential causal relationship between gut microbiota and 90 cardiovascular proteins. Using multi-cohort GWAS statistics of the gut microbiome and cardiovascular proteins, we identified 13 bacterial signatures that were causally associated with 11 cardiovascular proteins. Phylum *Euryarchaeota* has a high incidence and diversity in two body sites, the gut and oral cavity, as shown by Cai et al. Phylum *Euryarchaeota*, as an archaea may produce cancer-related metabolites that contribute to the human tumour microenvironment and carcinogenesis. This is consistent with our findings of a positive correlation between phylum *Euryarchaeota* and CA-125 (Cai et al., 2022). CSF-1 stimulates macrophage colonization and granulocyte function, and lowers blood cholesterol (Hume and MacDonald, 2012). He et al. found that order *Bacillales* lowers cholesterol (He et al., 2021). Unfortunately, this contradicts our results. Heat shock protein 27 (Hsp27), also known as heat shock protein beta-1 (HSPB1), is encoded by the HSPB1 gene. It has a molecular chaperone activity, contributes to heat resistance, inhibits apoptosis, and regulates cell development and differentiation (Van Montfort et al., 2001). Qiu et al. found that, after concurrent chemoradiotherapy of patients with non-small cell lung cancer, patients in the long-term progression-free survival group had increased faecal genus *Dorea* abundance (Qiu et al., 2022). Interestingly, we also found a positive causal relationship between genus *Dorea* and HSP-27 (β = 0.465). In a randomized controlled trial on the diet of patients with irritable bowel syndrome, serum proinflammatory IL-6 and IL-8 levels, and faecal abundance of *actinomycetes*, *bifidobacteria*, and *E. faecalis* were significantly lower in patients on the low-FODMAP diet (Hustoft et al., 2017). Coincidentally, Feng et al. reduced bacterial load with shen-ling-bai-zhu-san, which reduced bacterial abundance including *Actinobacteria* and inhibited interleukin (IL)-1β, IL-6, tumour necrosis factor-α, and IL-8 (Feng et al., 2020). In accordance with our results, a decrease in IL-8 may be partly caused by reduced abundance of phylum *Actinobacteria*. Many studies have reported that increased order *Enterobacteriales* may allow endotoxins to continuously enter circulation through an impaired intestinal barrier function, inducing a systemic inflammatory response to worsen the condition of patients with chronic kidney disease (Wu et al., 2021; Liu et al., 2022). Similarly, our study suggested that order *Enterobacteriales* was negatively correlated to plasma KIM-1 levels. Significantly elevated plasma levels of KIM-1 usually indicate early kidney injury. During this period, KIM-1 may be involved in early injury and repair of renal tubular epithelial cells and renal interstitial fibrosis through adhesion, clearance of apoptotic cells, and the immune response (Ichimura et al., 2012). Additionally, we found that phylum and class *Actinobacteria* was negatively correlated to LEP, a hormone produced mainly by adipocytes and intestinal epithelial cells in the small intestines, which regulates the energy balance by suppressing appetite and reducing fat storage in adipocytes (Machleidt and Lehnert, 2011). This is consistent with the findings of Yin et al. and Marques et al. who found that lysine restriction increases appetite by increasing the abundance of *Actinobacteria*, *Saccharibacteria*, and *Synergistetes* (Yin et al., 2017). Similarly, Marques et al. found lower levels of *Actinobacteria* spp. and *Prevotella*, but higher levels of *Bifidobacterium* and *Lactobacillus* in the intestinal flora of a high-fat-diet-induced obese rat model (Marques et al., 2016). These possible intermediates are caused by a decrease of LEP in plasma through *Actinobacteria* and *Bifidobacterium*. NT-proBNP is a hormone with an inactive N-terminal of 76 amino acids, which is cleaved from the molecule to release brain natriuretic peptide (Bhalla et al., 2004). Infusion of BNP at pathophysiological concentrations into hypertensive patients results in a progressive decrease in the left ventricular end-diastolic volume and left ventricular end-systolic volume as well as a reduction in cardiac preload (Atisha et al., 2004). Lv et al. found significant reductions in five bacterial species in the gut microbiota profile of early-onset preeclampsia women, including genus *Methanobrevibacter* (Lv et al., 2019). Thus, we hypothesized that plasma NT-proBNP decreases with the decrease in faecal genus *Methanobrevibacter*, which contributes to elevated blood pressure in patients. Jia et al. found an increase in the abundance of four bacteria, *Lactobacillus*, *Actinomyces*, *Peptostreptococcaceae*, and *Alloscardovia*, in the intestinal microbiota of intrahepatic cholangiocarcinoma patients (Jia et al., 2020). Furthermore, Liu et al. found that autocrine osteopontin promotes hepatic progenitor cell expansion and migration by binding to αv integrins and activating SRC activity to reduce membrane E-calmodulin and increase free cytoplasmic β-linked proteins. Moreover, this pathway plays a key role in the oncogenic transformation of hepatic progenitor cells (Liu et al., 2015). In combination with these previous studies, our findings led us to the hypothesis that the role of family *Peptostreptococcaceae* in the progression of tumours, including hepatocellular carcinoma, may be mediated through activation of SRC protein kinase, causing activation of downstream pathways. Tumour necrosis factor receptor 2 (TNFR2), also known as tumour necrosis factor receptor superfamily member 1B (TNFRSF1B) and CD120b, is one of two membrane receptors that bind tumour necrosis factor-α (TNFα) (Schall et al., 1990). In a study of the effect of intermittent fasting (IF) on diabetic retinopathy, Beli et al. found that the intestinal tract of IF mice had a reduction in both *Bacteroidetes* and *Verrucomicrobia* bacteria and that IF decreased retinal TNF-α expression compared with mice fed ad libitum (Beli et al., 2018). Therefore, understanding whether phylum and order *Verrucomicrobia* are also involved in reductions of TNF-α and its membrane receptor TNF-R2 may provide another novel insight into the causal relationship between *Verrucomicrobia* and TNF-R2. Sickle cell disease (SCD) is an inherited blood disorder that causes intravascular haemolysis, and slow blood flow can cause microthrombosis resulting in organ damage. In 14 healthy controls and 14 SCD subjects, severe ecological dysregulation was observed in SCD patients with many flora being abundant, including the family *Veillonellaceae* (Brim et al., 2021). t-PA is a protein involved in thrombus breakdown. It is a serine protease that catalyses conversion of fibrinogen to fibrinolytic enzymes, the main enzyme responsible for clot breakdown (Wardlaw et al., 2012). In agreement with our results, an increase in family *Veillonellaceae* positively led to an increase in plasma t-PA. Pindjakova et al. found that, compared with mice fed a hypercholesterolemic and proatherogenic diet, low-fat diet (Paigen diet)-fed mice, *Gammaproteobacteria*, *Delataproteobacteria*, and *Erysipelotrichia* were more abundant in obese mice fed a dietary, high-fat obesogenic, but non-inflammatory diet (Pindjakova et al., 2017). Unfortunately, we did not find any correlation between class *Erysipelotrichia* and VEGF-D for the time reported. We screened 11 plasma cardiovascular proteins and found 14 causal relationships between gut microbiota and these proteins.

In fact, in addition to the effect of gut microbiota on cardiovascular disease, we found that gut microbiota influences markers for tumour progression, endocrine metabolism, and renal organism on the basis of previous studies. However, it is undeniable that our study has certain shortcomings. Due to the limitations of data from gut microbes studies, the bacterial taxa mentioned in this article were only analysed at the genus and above level and not at the level of more specific species or strains. Our sample sizes of gut microbiota and cardiovascular proteins were not very large compared with some MR analyses with different aims. Moreover, the number of SNPs representative of each microbiota was relatively small. Further large-scale RCT clinical trials and studies of biological mechanisms are needed.

Overall, we comprehensively assessed the potential causal relationship between gut microbiota and cardiovascular protein expression. Combined with other studies, we partially confirmed our conclusions. Therefore, this study may provide new insights into microbially mediated alterations in cardiovascular protein levels.

## Data availability statement

The datasets presented in this study can be found in online repositories. The names of the repository/repositories and accession number(s) can be found in the article/**Supplementary Material**.

## Ethics statement

Studies involving human participants were reviewed and approved, and all studies were approved by their respective institutional review boards. No new data were collected and no new institutional review boards’ approval was required.

## Author contributions

All authors contributed to the work presented in this paper. Conceptualisation, WZ and ZW. Resources, visualisation, and analysis, SZ, FZ and JD. writing—original draft preparation, WZ and SZ. writing—editing, ZW. supervision, ZW. project administration, WZ and ZW. funding acquisition, FZ and ZW. All authors approved the submitted version.

## Funding

This work was supported by the National Natural Science Foundation of China (No. 81601692 and 81901969), the Technology Research from the Department of Education of Liaoning Province (No. JCZR2020013), 345 Talent Project of Shengjing hospital of China Medical University.

## Acknowledgment

We thank Mitchell Arico from Liwen Bianji (Edanz) (https://www.liwenbianji.cn) for editing the language of a draft of this manuscript.

## Conflict of interest

The authors declare that the research was conducted in the absence of any commercial or financial relationships that could be construed as a potential conflict of interest.

## Publisher’s note

All claims expressed in this article are solely those of the authors and do not necessarily represent those of their affiliated organizations, or those of the publisher, the editors and the reviewers. Any product that may be evaluated in this article, or claim that may be made by its manufacturer, is not guaranteed or endorsed by the publisher.
