# Supplementary material for: Causal relationship between gut microbes and cardiovascular protein expression
Source: Front Cell Infect Microbiol. 2022 Dec 5;12:1048519. doi: 10.3389/fcimb.2022.1048519 (PMC9760811; doi:10.3389/fcimb.2022.1048519)

Supplemental Figure 1. MR leave-one-out sensitivity analysis for 14 causal associations from 13 microbiota and 11 cardiovascular proteins.


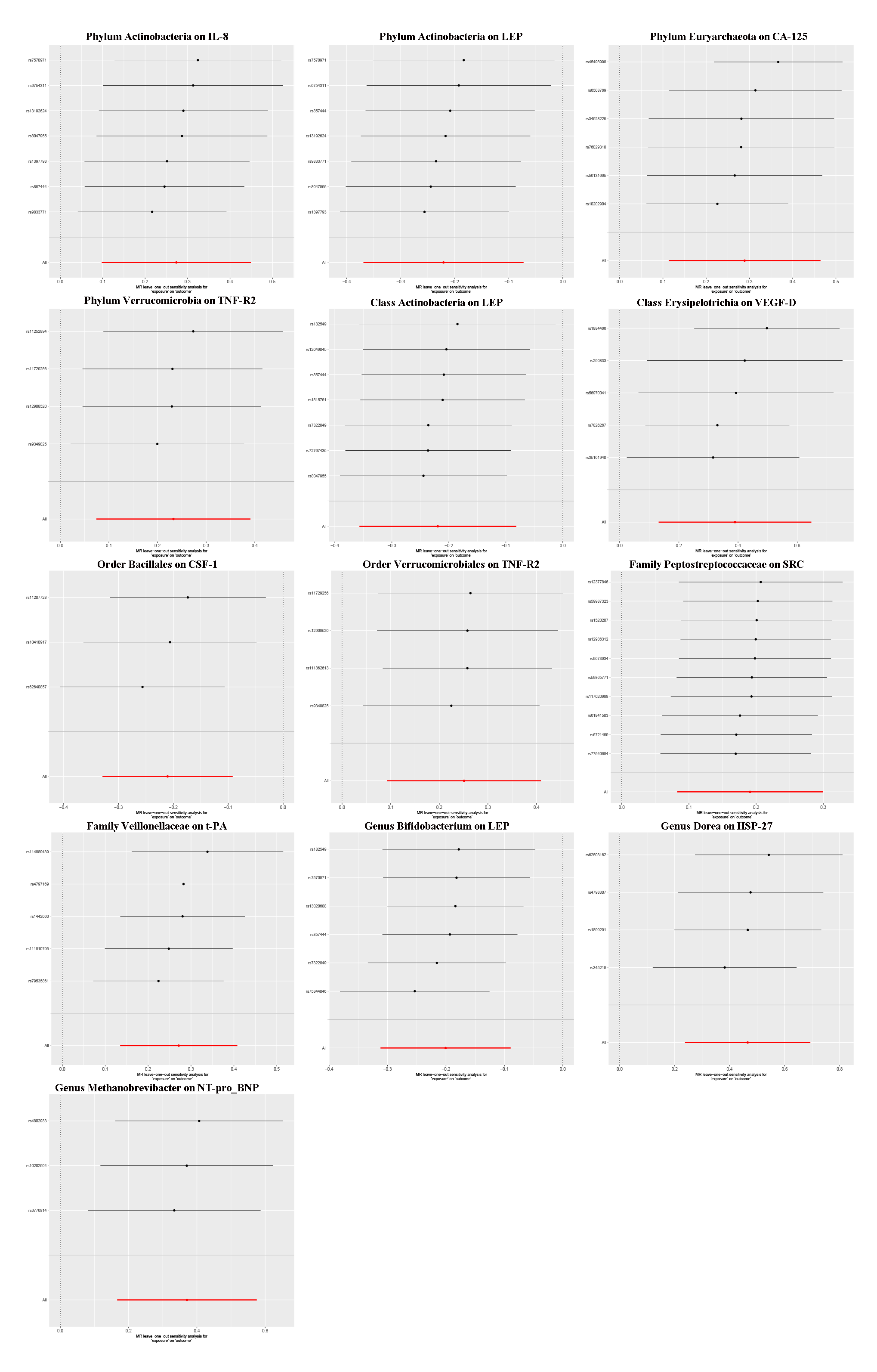

Supplement: Supplementary file 5 [file Table_5.docx]
